# Evaluation of Potential Complications of Interstitial Lung Disease Associated With Antiandrogens Using Data From Databases Reporting Spontaneous Adverse Effects

**DOI:** 10.3389/fphar.2021.655605

**Published:** 2021-06-09

**Authors:** Hideki Nawa, Takahiro Niimura, Hirofumi Hamano, Kenta Yagi, Mitsuhiro Goda, Yoshito Zamami, Keisuke Ishizawa

**Affiliations:** ^1^ Department of Pharmacy, Faculty of Pharmacy, Shujitsu University, Okayama, Japan; ^2^ Department of Clinical Pharmacology and Therapeutics, Graduate School of Biomedical Sciences, Tokushima University, Tokushima, Japan; ^3^ Clinical Research Center for Developmental Therapeutics, Tokushima University Hospital, Tokushima, Japan; ^4^ Department of Pharmacy, Tokushima University Hospital, Tokushima, Japan

**Keywords:** interstitial lung disease, antiandrogen, bicalutamide, flutamide, prostate cancer

## Abstract

From 2002 to 2018, the number of patients with prostate cancer significantly increased from 679,023 to 1276,106 worldwide. Total prostatectomy (including robot-assisted prostatectomy), radiation therapy, and pharmacological treatment are commonly used to treat prostate cancer. The Chief of the Pharmaceutical Safety Division, that is, the Federation of Pharmaceutical Manufacturers’ Associations of Japan (FPMAJ), recently called for the revision of package inserts for ethical drugs. However, the pathogenesis of interstitial lung disease (ILD), a serious drug-induced adverse effect, remains unclear. Moreover, there have been no large-scale evaluations of potential complications associated with currently used antiandrogens, which are commonly employed to treat prostate cancer. Hence, ILD, as an adverse event, remains poorly understood. Therefore, we conducted a survey of reports in the Japanese Adverse Drug Event Report (JADER) database to investigate the potential association between the reporting of ILD and antiandrogen drug use in clinical practice. The occurrence of ILD was investigated by evaluating the relationship between antiandrogen drug use and ILD. Adverse event signals were detected with reporting odds ratios (RORs), using data from the JADER and FDA Adverse Event Reporting System (FAERS) databases, for the analysis of post-marketing adverse event reports. The JADER was used to examine the time profile of adverse event occurrence for each drug, whereas the FAERS was used to screen cases of unknown adverse events and analyze their trends of occurrence. The analysis of data from both databases revealed the 95% confidence interval lower limits of ROR for bicalutamide and flutamide to be > 1, and adverse event signals were detected following the use of either drug. While caution should be exercised for drugs that are new to the market, we conclude that drugs with similar therapeutic effects that have been in use for a long period should also be re-examined for potential adverse events.

## Introduction

From 2002 to 2018, the number of patients with prostate cancer significantly increased from 679,023 to 1276,106 worldwide (Parkin et al., 2005; Torre et al., 2015; Bray et al., 2018). Total prostatectomy (including robot-assisted prostatectomy), radiation therapy, and pharmacological treatment are commonly used to treat prostate cancer. Additionally, the efficacy and safety of various drug therapies including hormones and antiandrogenic drugs, as well as chemotherapeutics, have been investigated in various studies; the results have prompted the clinical use of these therapies in an effort to improve prostate cancer treatment (National Comprehensive Cancer Network, 2018). Meanwhile, androgen receptor antagonists including enzalutamide and apalutamide have been recently developed. The major adverse effects of these drugs include fatigue, skin rash, hypothyroidism, pruritus, and weight loss. Convulsive seizures, heart problems, and severe skin disorders have been reported as serious adverse effects (Astellas, 2018; Janssen Pharmaceutical, 2019). These agents inhibit not only the binding of androgens to androgen receptors but also their nuclear translocation, thereby suppressing the growth of androgen-dependent tumors, leading to the development of castration-resistant, castration-free, distant metastasis-free, and androgen-dependent tumors (Clegg et al., 2012). Therefore, these antagonists have been indicated for patients with prostate cancer, and they are expected to improve treatment efficacy (Clegg et al., 2012; Smith et al., 2018).

These drug therapies may elicit an array of adverse effects including interstitial lung disease (ILD), which is a serious drug-induced adverse effect with no known mechanism of onset. The average survival time of patients with ILD is 28–52 months from the confirmation of diagnosis, according to European and American reports, and 61–69 months from the time of initial diagnosis, according to Japanese reports (The Japanese Respiratory Society, 2020). An investigation based on the opinion of an expert committee of the Pharmaceutical and Consumer Health Bureau of the (Ministry of Health, Labor, and Welfare, 2019)of Japan, on November 15, 2019, reported serious adverse effects associated with administration of Erleada® (apalutamide) 60 mg tablet, launched in May 2019, wherein the causal relationship with ILD could not be ruled out. However, one death, following the administration of Erleada® (60 mg tablets), was associated with an undeniable causal relationship to ILD. In response, the Ministry of Health, Labour and Welfare’s Pharmaceutical Affairs and Consumer Health Bureau instructed The Chief of the Pharmaceutical Safety Division, that is, the Federation of Pharmaceutical Manufacturers’ Associations of Japan (FPMAJ), to revise the package inserts of ethical drugs associated with serious adverse effects (Director of Office of Safety Division, Pharmaceutical Safety and Environmental Health Bureau, 2019). Considering that the incidence of antiandrogen-induced ILD is not known, any drug suspected to induce lung injury should be discontinued promptly, regardless of the adverse event severity. If the treatment cannot be discontinued, the patient should be switched to another type of drug that is less likely to have the same adverse events. However, antineoplastic therapy should not be resumed until the patient’s lung injury has improved (Kohno et al., 1993; Ohnishi et al., 2003; The Japanese Respiratory Society, 2020).

The resulting changes included the addition of “interstitial lung disease” to the “serious adverse reactions” section of the package inserts for enzalutamide and apalutamide, with the revision of the associated warnings. However, there have been no large-scale evaluations of the potential complications of enzalutamide, apalutamide, and other antiandrogens that are currently used. Furthermore, the circumstances associated with ILD occurrence remain unclear. Although the cause of ILD is unknown, diverse genetic backgrounds, chronic inflammations, and repeated alveolar epithelial damages induced by environmental factors have been proposed as causative factors. Smoking is considered the most important “risk factor” that may not be a direct cause of ILD but has an indirect effect, particularly in patients with idiopathic pulmonary fibrosis (IPF). Dust exposure, which is an obvious cause, is an exclusionary condition for IPF. Although there are several reports on genetic polymorphisms that are highly responsive to environmental factors, including these risk factors, hereditary interstitial pneumonia is distinguished as familial pulmonary fibrosis. Abnormalities in genes related to surfactant proteins and their release mechanisms are associated with familial pulmonary fibrosis (Raghu et al., 2011). It would be useful to investigate both newly approved drugs and older drugs using data from the Japanese Adverse Drug Event Report (JADER) database and FDA Adverse Event Reporting System (FAERS) for detecting rare adverse events such as ILD (Fukazawa et al., 2018; Raschi et al., 2021). Therefore, in the current study, we investigated the potential association between the reporting of ILD and antiandrogen drug usage in clinical practice by conducting a survey of reports in the JADER database, published by the Pharmaceuticals and Medical Devices Agency. Data from the JADER and FAERS, published by the United States Food and Drug Administration (FDA), were used to determine the reporting odds ratios (RORs) to detect adverse event signals for the evaluation of relationships between antiandrogens and ILD.

## Materials and Methods

Data from the JADER database were downloaded from the Pharmaceuticals and Medical Devices Agency (PMDA) website (https://www.pmda.go.jp/, accessed on March 4, 2020). The JADER database consists of four files: DEMO, DRUG, REAC, and HIST. The DEMO file contains basic information about patients, including sex, age, and weight. The DRUG file contains information about the drug, such as the generic name, route of administration, and start and end dates of administration. The REAC file contains the name of adverse events, their outcomes, and the date of occurrence. The HIST file contains information on the primary disease of patients (Supplementary Table S1, Supplementary Table S2 **)**.

The FAERS database, downloaded from the FDA website (http://www.fda.gov/ accessed on January 8, 2020) comprised seven files, namely, DEMO, DRUG, REAC, OUTC, RPSR, INDI, and THER. The DEMO file contains basic information about patients, including sex, age, date of adverse event, and country of occurrence of the adverse event. The DRUG file contains information such as the name of drugs, route of administration, and dose of drugs. The REAC file contains the name of adverse events; the OUTC file contains details of case outcomes; the RPSR file contains details of the source of adverse events; the INDI file contains details of indications; and the THER file contains information about the initiation date, end date, and treatment duration. The analysis period for this study was from April 2004 to December 2019 for the JADER and from January 2004 to December 2019 for the FAERS (Supplementary Table S1, Supplementary Table S2).

The names of adverse events were extracted from the ICH Medical Dictionary for Regulatory Activities (MedDRA) Ver. 22.1J and from standardized MedDRA queries (SMQs) for ILD. In this study, the basic term of ILD was set as “preferred terms” (PT) to narrow the scope terms. Antiandrogen drugs, such as enzalutamide, apalutamide, bicalutamide, flutamide, chlormadinone acetate, and abiraterone acetate, were included in the analysis, and so were patients’ age and sex. The timing of adverse drug events in patients reporting adverse reactions was determined using a log-rank test with EZR (Easy R), freely available statistical software (Inaba et al., 2019).

### Methods of Analysis

To evaluate the signals of ILD, the ROR and 95% confidence interval (CI) for ILD were calculated using the following formula, with the ROR adjusted for age, sex, and reporting year:

$$ROR=\frac{a/c}{b/d},95\%CI=exp\left\{\log\left(ROR\right)\pm 1.96\sqrt{\frac{1}{a}+\frac{1}{b}+\frac{1}{c}+\frac{1}{d}}\right\}$$

where, *a* represents cases that belong to the group identified as ILD; *b* represents cases that did not belong to the group but were identified as ILD; *c* represents cases that belong to the group and were not identified as ILD; and *d* represents cases that did not belong to the group and were not identified as ILD (Rothman et al., 2004; Kanda, 2013). The cases presented were not necessarily only patients with prostate cancer, but also patients taking targeted therapy drugs. The JADER database was created using Access 2016 (Microsoft). NaviCat for SQLite (Premium Soft) was used to construct the FAERS database. The ROR was calculated using a 2 × 2 contingency table divided according to the use of drug and occurrence of adverse events. As the FAERS and JADER are spontaneous adverse event reporting databases, and there was no control group, the obtained odds ratio was distinguished from normal odds ratio as an ROR. A signal was considered to be present if the lower limit of the 95% CI of the calculated ROR was >1. We also detected the signal for cases belonging to the age group >60 years. A male signal was also detected. The drugs causing ILD were identified as amiodarone, bleomycin, cyclophosphamide, gefitinib, and methotrexate with reference to previous studies (Table 1) (Matsuno, 2012; Skeoch et al., 2018). The target for comparison in the ROR calculation was all drugs recorded in the database.

**TABLE 1:** Major interstitial lung disease-inducing drugs.

Drug	Total	Cases	ROR (95% CI)
JADER	611,336	33,099	—
Amiodarone	3,180	798	6.0 (5.5–6.5)
Bleomycin	612	133	4.8 (4.0–5.9)
Cyclophosphamide	10,833	758	1.3 (1.2–1.4)
Gefitinib	3,038	1,317	13.9 (12.9–14.9)
Methotrexate	29,966	2,460	1.6 (1.5–1.7)
FAERS	11,448,913	66,335	—
Amiodarone	53,866	3,353	11.9 (11.5–12.4)
Bleomycin	6,820	729	20.8 (19.2–22.4)
Cyclophosphamide	114,296	3,488	5.6 (5.5–5.8)
Gefitinib	6,788	514	14.2 (12.9–15.5)
Methotrexate	308,015	6,138	3.7 (3.6–3.8)

## Results

The total number of JADER reports was 611,336 and the number of reported ILD cases was 33,099. No significant characteristics were detected in the patient outcomes of the JADER and FAERS (Table 2). The medications causing ILD were similar in both JADER and FAERS (Table 1). For all enrolled patients, the JADER had reports of 8,469 patients with prostate cancer and 7,394 patients with enlarged prostate, of which 523 were duplicates, and the FAERS had reports of 86,004 patients with prostate cancer and 21,501 patients with enlarged prostate, of which 592 were duplicates. For patients with ILD, the JADER had reports of 730 patients with prostate cancer and 622 patients with enlarged prostate, of which 44 were duplicates, and the FAERS had reports of 1,045 patients with prostate cancer and 305 patients with enlarged prostate, of which 25 were duplicates (data not shown). In addition, in a certain number of patients, both benign prostatic hyperplasia and prostate cancer were reported as the primary cause of ILD. The ROR (95% CI) in the JADER for the tested antiandrogen drugs is listed in Table 2. The 95% CI lower limit of the ROR for bicalutamide and flutamide was >1, and adverse event signals were detected in both cases (Table 3). Interstitial lung disease was reported for all six antiandrogenic agents (Table 4), with the lowest number of reports (1 report) for apalutamide and the highest number for bicalutamide (208). Based on the reported imbalance, using ROR (the index value of signals related to adverse effects and drugs), we analyzed the characteristics of drugs with high ROR values by age and sex. The ROR of the six antiandrogens investigated in this study is provided in Table 5. The 95% CI lower limit of ROR for bicalutamide and flutamide was >1, and adverse event signals were detected when the two drugs were used (Table 5). As prostate cancer occurs in males, we analyzed the data of men, and as it has been reported that the PSA level at the age of 60 years can be used to predict subsequent prostate cancer diagnosis, metastasis, and death from prostate cancer, we analyzed data of patients in the age range of 60 years and above (Vickers et al., 2010; Carlsson et al., 2014).

**TABLE 2:** Outcomes in patients with interstitial lung disease.

JADER	—
Total ILD cases	33,099
Deaths	5,489
Unrecovered	2,745
Recovered with sequelae	567
Remission	11,353
Recovery	8,446
Unknown	4,683
FAERS	—
Total ILD cases	66,335
Deaths	18,473
Life-threatening	7,025
Hospitalization (initial or prolonged)	36,417
Disability	2,621
Congenital anomaly	212
Required intervention to prevent permanent impairment/damage	548
Additional serious medical events	35,996

**TABLE 3:** Number of reports and the reporting odds ratios by antiandrogen drugs.

Drug	Total	Cases	ROR (95% CI)
JADER	611,336	33,099	–
Enzalutamide	1,545	47	0.5 (0.4–0.7)
Apalutamide	37	2	1.0 (0.2–4.2)
Bicalutamide	2,303	394	3.6 (3.3–4.1)
Flutamide	471	42	1.7 (1.2–2.4)
Chlormadinone acetate	590	33	1.2 (0.9–1.8)
Abiraterone acetate	1,837	56	0.5 (0.4–0.7)
FAERS	11,448,913	66,335	–
Enzalutamide	42,508	106	0.4 (0.4–0.5)
Apalutamide	1,114	4	0.6 (0.2–1.7)
Bicalutamide	10,594	388	6.6 (5.9–7.3)
Flutamide	1,039	40	6.9 (5.0–9.4)
Chlormadinone acetate	429	24	10.2 (6.7–15.4)
Abiraterone acetate	22,185	138	1.0 (0.9–1.3)

**TABLE 4:** Medians for the time-to-onset of interstitial lung disease preferred terms (PTs) in the JADER database.

Drug	Number of reports	Average (day)	Time-to-onset (day) median (25–75%)
Enzalutamide	27	97.7	27.0 (14.5–129)
Apalutamide	1	64.0	64.0 (64–64)
Bicalutamide	208	306.0	89.5 (32–357)
Flutamide	29	330.9	117.0 (62–463)
Chlormadinone acetate	21	167.2	78.0 (41–227)
Abiraterone acetate	33	189.3	123.0 (56–280)

**TABLE 5:** Characteristics of potential complicating factors for interstitial lung disease preferred terms (PTs) in the JADER database.

•Characteristic	Total	a/c	b/d
Aged ≥60 years			
Enzalutamide	1,231	40/1,191	24,785/323,644
Apalutamide	31	2/29	24,823/324,806
Bicalutamide	2068	350/1718	24,475/323,117
Flutamide	418	41/377	24,784/324,458
Chlormadinone acetate	448	33/415	24,792/324,420
Abiraterone acetate	1,327	49/1,278	24,776/323,557
Male			
Enzalutamide	1,542	47/1,495	19,835/276,499
Apalutamide	37	2/35	19,800/277,959
Bicalutamide	2,295	392/1903	19,490/276,091
Flutamide	468	42/426	19,840/277,568
Chlormadinone acetate	469	35/434	19,847/277,560
Abiraterone acetate	1836	56/1780	19,826/276,214

a Represents cases that belong to the group identified as ILD.

b Represents cases that did not belong to the group but were identified as ILD.

c Represents cases that belong to the group and were not identified as ILD.

d Represents cases that did not belong to the group and were not identified as ILD.

The total number of reports in the FAERS database was 11,448,913, of which 66,335 (0.58%) were ILD cases. The ROR (95% CI) in the FAERS for the six antiandrogens investigated in this study are presented in Table 3. The 95% CI lower limit of the ROR for bicalutamide and flutamide was >1, and thus, adverse event signals were detected for the two drugs (Table 3).

## Discussion

This study was initiated following the inclusion of ILD as a serious adverse effect in the package inserts of enzalutamide and apalutamide (The Japanese Respiratory Society, 2020). Other antiandrogens were also included in the evaluation of potential complication analysis of ILD. The drugs were selected based on the possibility that antiandrogenic effects may be involved in the development of adverse effects. In both JADER and FAERS, the outcome of patients with ILD based on patient background data was mild or required hospitalization, but not death. Moreover, drugs that have been reported to cause ILD showed a high signal in both JADER and FAERS. Furthermore, drugs such as amiodarone, bleomycin, and gefitinib, which have been reported to cause ILD, showed high signals in both JADER and FAERS (Matsuno, 2012; Skeoch et al., 2018) (Table 1). In the JADER and FAERS, all drugs known to cause drug-induced ILD showed a high signal. In addition, prostate cancer and benign prostatic hyperplasia were found to be the primary causes of ILD in both databases. The results of the present study are supported by the fact that sex steroid hormones have been implicated in the pathogenesis of ILD and pulmonary fibrosis (Verma et al., 2011). However, the pathogenesis of drug-induced lung injury remains unclear, and various mechanisms have been considered (Zitnik and Matthay, 1998; Fraser et al., 1999; Inoue et al., 2003).

Although the causes of ILD are unknown, diverse genetic background, chronic inflammation, and repeated alveolar epithelial damage induced by environmental factors have been proposed as causative factors. Interstitial lung disease has several causes, including most connective tissue diseases, occupational exposures, and many drugs (Skeoch et al., 2018; Matsuno, 2012).

All nonsteroidal antiandrogens share the same antiandrogenic effect. Moreover, although the mechanisms of action of nonsteroidal antiandrogens can vary, all antiandrogenic drugs, including bicalutamide, flutamide, and nilutamide, as well as the more recently licensed enzalutamide and apalutamide, appear to be similar in that they inhibit the binding of androgens to androgen receptors (Astellas, 2018; Janssen Pharmaceutical, 2019). This may lead to the development of ILD. Bicalutamide and flutamide are nonsteroidal antiandrogenic agents. The difference in the effect of sexual function may have some influence as to why the signal appeared only for bicalutamide and flutamide (Mahler et al., 1998; Goa Spencer, 1998). The antiandrogenic effect causes repeated damage to alveolar epithelial cells and abnormalities in their repair and healing processes and may be involved in genetic abnormalities involved in alveolar epithelial cell function. Hence, there is a concern that the incidence of ILD may increase with repeated administrations of flutamide and bicalutamide. Indeed, currently, the package inserts of bicalutamide and flutamide include a warning that they cause interstitial pneumonia in less than 0.1% of patients (Nippon Kayaku Co., Ltd., 2014; AstraZeneca, 2016). Although the reason for the lack of signals for enzalutamide and apalutamide is unclear, the results obtained in this study suggest that both drugs, bicalutamide and flutamide, bind to androgen and inhibit androgen binding. Thus, there is a concern that the incidence of ILD may increase as the number of doses increases.

Although the timing of adverse effect occurrence varies for each antiandrogenic drug, the reported average duration for bicalutamide and flutamide use, before ILD development, is approximately 1 year, suggesting the importance of being cautious to the occurrence of adverse effects during their long-term use. Moreover, considering the specific warnings for enzalutamide and apalutamide, physicians must proceed with caution when prescribing these drugs to avoid potential emergence of ILD. Specifically, we believe, that caution should be exercised for approximately 1 year, depending on the drug.

A comparison of data from the FAERS and JADER is helpful for evaluating the differences based on race, as it allows comparisons between data of countries around the world and those of Japan. A comparison of results from the FAERS and JADER provided complimentary adverse event information. In this study, we found differences in the occurrence of ILD with the use of different antiandrogen drugs. However, a lack of detailed information regarding the patient background (e.g., symptoms and medication status) in the JADER and FAERS precluded analysis based on disease severity. That is, the signals detected in this study suggested a statistical association between the drugs and adverse events; however, they could not indicate a causal relationship. Therefore, a more detailed evaluation of the signals would be required in future studies. It should be particularly noted that the lack of signals in certain conditions in this study, does not imply the absence of an association between the drug and adverse events. A limitation of this study is several biases exist due to the data set created with reported cases. Therefore, it should be noted that factors that affect the results, such as concomitant medications, have not been reported in their entirety. As the JADER and FAERS are composed of reports, the population of patients using the drugs is not known. Therefore, as a substitute, patients with reports of adverse events other than those of interest are treated as the population. Even if the number of FAERS reports is limited to data from Japan and the United States, the available Japanese data account for less than 5% of the total United States data. In fact, as there are reports from countries other than Japan, the amount of Japanese-specific data in FAERS is small; hence, the overlap of data between the FAERS and JADER will not have a significant effect on the analysis results. Therefore, we believe that the duplicates in the FAERS and JADER do not likely have a significant effect on the overall analysis results (Nomura et al., 2015).

The results of this study suggest that caution should be exercised not only for drugs that have been in the market for a relatively short period, but also for those that have been in use for a long time if they induce similar therapeutic effects. We believe that the use of ROR is effective for determining whether the frequency of adverse drug reactions is high; however, future studies are warranted to examine whether there is an actual causal relationship between a drug and adverse drug reactions. In particular, analysis should be conducted from the perspective of ILD and antiandrogen drugs.

## Data Availability

The original contributions presented in the study are included in the article/Supplementary Material, further inquiries can be directed to the corresponding author.
