# Supplementary material for: Evaluation of Potential Complications of Interstitial Lung Disease Associated With Antiandrogens Using Data From Databases Reporting Spontaneous Adverse Effects
Source: Front Pharmacol. 2021 Jun 9;12:655605. doi: 10.3389/fphar.2021.655605 (PMC8220081; doi:10.3389/fphar.2021.655605)
Supplement: Supplementary file 1 [file DataSheet1.docx]

Supplementary Material

**Table S1.** Patient background (Only those with data)

| JADER |  |
| --- | --- |
| Male | 297,876 |
| Female | 285,736 |
|  |  |
| Aged < 10 years | 22,530 |
| 10≦ aged <20 years | 16,351 |
| 20≦ aged <30 years | 18,875 |
| 30≦ aged <40 years | 31,945 |
| 40≦ aged <50 years | 43,400 |
| 50≦ aged <60 years | 71,250 |
| 60≦ aged <70 years | 127,390 |
| 70≦ aged <80 years | 141,106 |
| 80≦ aged <90 years | 71,403 |
| Aged > 90 years | 9,761 |
|  |  |
| Submitter |  |
| Physician | 437,254 |
| Pharmacist | 52,583 |
| Other health-professional | 21,931 |
| Lawyer | 74 |
| Consumer | 15,228 |
|  |  |
|  |  |
| FAERS |  |
| Male | 3,957,589 |
| Female | 6,326,525 |
|  |  |
| Aged < 10 years | 196,613 |
| 10≦ aged <20 years | 329,413 |
| 20≦ aged <30 years | 515,199 |
| 30≦ aged <40 years | 695,481 |
| 40≦ aged <50 years | 990,517 |
| 50≦ aged <60 years | 1,471,152 |
| 60≦ aged <70 years | 1,563,804 |
| 70≦ aged <80 years | 1,165,302 |
| 80≦ aged <90 years | 542,449 |
| Aged > 90 years | 106,973 |
|  |  |
| Reporter Country |  |
| United States | 7,667,351 |
| Japan | 104,386 |
| United Kingdom | 111,226 |
| France | 90,977 |
| Germany | 74,711 |
| Canada | 50,959 |
| Brazil | 35,057 |
| Italy | 24,410 |
|  |  |
| Submitter |  |
| Physician | 2,618,231 |
| Pharmacist | 756,412 |
| Other health-professional | 2,038,889 |
| Lawyer | 200,769 |
| Consumer | 5,200,170 |

**Table S2**. Involvement of pharmaceuticals in ILD (Only those with data)

| JADER |  |
| --- | --- |
| Enzalutamide |  |
| suspect drug | 33 |
| Concomitant | 14 |
| Interacting | 0 |
| Apalutamide |  |
| Suspect Drug | 2 |
| Concomitant | 0 |
| Interacting | 0 |
| Bicalutamide |  |
| Suspect Drug | 277 |
| Concomitant | 113 |
| Interacting | 5 |
| Flutamide |  |
| Suspect Drug | 22 |
| Concomitant | 19 |
| Interacting | 1 |
| Chlormadinone Acetate |  |
| Suspect Drug | 4 |
| Concomitant | 30 |
| Interacting | 1 |
| Abiraterone Acetate |  |
| Suspect Drug | 40 |
| Concomitant | 16 |
| Interacting | 0 |
| Amiodarone |  |
| suspect drug | 766 |
| Concomitant | 16 |
| Interacting | 0 |
| Bleomycin |  |
| Suspect Drug | 127 |
| Concomitant | 6 |
| Interacting | 0 |
| Cyclophosphamide |  |
| Suspect Drug | 473 |
| Concomitant | 287 |
| Interacting | 1 |
| Gefitinib |  |
| Suspect Drug | 1,293 |
| Concomitant | 34 |
| Interacting | 0 |
| Methotrexate |  |
| Suspect Drug | 2,134 |
| Concomitant | 343 |
| Interacting | 1 |
| FAERS |  |
| Enzalutamide |  |
| Primary Suspect Drug | 68 |
| Secondary Suspect Drug | 37 |
| Concomitant | 16 |
| Interacting | 0 |
| Apalutamide |  |
| Primary Suspect Drug | 4 |
| Secondary Suspect Drug | 3 |
| Concomitant | 0 |
| Interacting | 0 |
| Bicalutamide |  |
| Primary Suspect Drug | 1 |
| Secondary Suspect Drug | 158 |
| Concomitant | 128 |
| Interacting | 2 |
| Flutamide |  |
| Primary Suspect Drug | 1 |
| Secondary Suspect Drug | 7 |
| Concomitant | 23 |
| Interacting | 1 |
| Chlormadinone Acetate |  |
| Primary Suspect Drug | 0 |
| Secondary Suspect Drug | 5 |
| Concomitant | 17 |
| Interacting | 1 |
| Abiraterone Acetate |  |
| Primary Suspect Drug | 97 |
| Secondary Suspect Drug | 27 |
| Concomitant | 21 |
| Interacting | 0 |
| Amiodarone |  |
| Primary Suspect Drug | 709 |
| Secondary Suspect Drug | 1,592 |
| Concomitant | 370 |
| Interacting | 11 |
| Bleomycin |  |
| Primary Suspect Drug | 195 |
| Secondary Suspect Drug | 473 |
| Concomitant | 65 |
| Interacting | 2 |
| Cyclophosphamide |  |
| Primary Suspect Drug | 417 |
| Secondary Suspect Drug | 2,296 |
| Concomitant | 842 |
| Interacting | 12 |
| Gefitinib |  |
| Primary Suspect Drug | 447 |
| Secondary Suspect Drug | 133 |
| Concomitant | 7 |
| Interacting | 1 |
| Methotrexate |  |
| Primary Suspect Drug | 1,183 |
| Secondary Suspect Drug | 2,924 |
| Concomitant | 1,653 |
| Interacting | 41 |
